# Upon the Modifications Which Take Place in the Spinal Cord, Occasioned by the Section of the Nerves of a Limb

**Published:** 1870-02

**Authors:** A. Vulpian


### UPON THE MODIFICATIONS WHICH TAKE PLACE IN THE SPINAL CORD, OCCASIONED BY THE SECTION OF THE NERVES OF A LIMB.

By A. VULPIAN.

Translated for the EXAMINER, *from Archives de Physiologie Normale et Pathologique.*

I have described, in a paper published last year, the modifications which take place in the spinal cord, following the amputation of one of the inferior members. In two cases, where I was able to examine the spinal nerve, a long time after the amputation of a limb, I have observed a diminution of volume in the corresponding half of the cord, especially in the region of this organ, associated with the origin or termination of the nerves, destined for the amputated part.

Some months afterward, M. W. H. Dickinson published * the results, which he had obtained during previous years, from examinations of the spinal cord, in similar conditions.

M. Dickinson mentions four cases; but in one of these, where he encountered an amputation of the thigh, the spinal cord could not be examined. He was obliged to confine his investigations to the sciatic nerve of the stump; and he ascertained the disappearance of a considerable number of nervous fibres, although the nerve had retained its ordinary aspect and its normal size. M. Dickinson, then, has studied the spinal cord, in three cases of amputation. In the first case, he examined the cord of a man, 74 years old, who had submitted to amputation of the left thigh, at the reunion of the upper and middle thirds, when he was 21 years of age, that is 53 years before his death. Here again, in the sciatic nerve of the side operated upon, the disorganization was so complete, that the nervous structure could scarcely be recognized, and the muscles of the stump were greatly atrophied. There was only a very slight diminution of the volume of the gray substance of the left side. At the level, and throughout the length, of the dorso-lumbar enlargement, the left posterior fascia was, in every way, perceptibly reduced, and especially so, near the gray commissure. This alteration appeared in the whole extent of the dorsal region. The connective tissue of this fascia appeared thicker than in the posterior fascia of the opposite side. Otherwise, there was no alteration of other parts of the cord, and the gray substance, on the right and the left sides, offered the same histological characteristics. The posterior roots of the left side, at the level of the inferior part of the cervical region, for a distance of two inches along the cord, presented a smaller volume than the corresponding roots of the right side. A considerable number of nervous fibres were much atrophied.

There was a similar alteration in the anterior roots of the same side, but in a less degree. In the cervical enlargement,

* On the Changes in the Nervous System, which Follow the Amputation of the Limbs.—*Journal of Anatomy and Physiology.* By G. M. Humphrey and W. Turner. November, 1868, p. 88.

the left posterior fascia was nearly one-third smaller than that of the right side, and there was visible a *train* of connective tissue, almost destitute of nervous fibres, extending from the surface of the cord toward the gray commissure, and separating *the* posterior fascia in two parts, nearly equal to each other. There was also a slight diminution of the volume of the gray substance, on this side, without perceptible histological modification. The alteration extended into the bulb, where it occupied only the posterior pyramid; beyond that could not be traced.

Finally, the third case has been furnished by another pensioner of Greenwich (age not given), who, in consequence of an accident, had suffered amputation of the right fore-arm, in 1845. He died in 1867, 22 years after the operation. Here, again, M. Dickinson has described quite evident atrophy of the corresponding posterior fascia (right side), in the entire length of the cervical enlargement. This fascia, as in the preceding case, was reduced to two-thirds of its normal volume. All the gray substance of this side was slightly diminished in size, without histological alteration.†

If the results obtained by M. Dickinson are compared with those given in my work, there will be found marked difference, as he himself has said. In cases which I have studied, the anterior fascia was especially atrophied. This fascia appears to have experienced no alteration of size, in the cords examined by M. Dickinson. On the contrary, the atrophy was limited, almost exclusively, to the posterior fascia of the side corresponding to the amputated member. Our descriptions invariably agree regarding the decreased dimensions of the gray substance, on the side of the operation. I have again examined the spinal cord, of which there was doubt, in my second observation, and I am satisfied that the description, as I have given it, is correct. Meanwhile, I have, perhaps, not stated with sufficient clearness, that the posterior fascia, in this instance, had not preserved its normal dimensions, and that it

† The work of M. Dickinson is accompanied with plates, which afford an exact idea of the modifications arising in the spinal cord, in these cases.

was much decreased in volume. This will be evident upon a glance at the figures which represent this nerve (1868, pl. 11, $B^1$ $B^2$).

At present, it is not possible for me to explain satisfactorily, the difference existing between the alterations described by M. Dickinson and those witnessed by myself; as observations increase, we shall understand these dissimilar conditions.

Since the publication of my paper upon this subject, I have had two opportunities of examining the spinal cord of persons who had submitted to the disarticulation or the amputation of a lower limb. I will give the results obtained in these two cases.

Obs. I. Mr. V., after the battle of Magenta, 1859, suffered a disarticulation of the left thigh. The operation was performed by Jules Roux. Ten years after (1869), the man died of phthisis pulmonalis, at the hospital, Lariboisiére, in charge of M. Oulmont. He was then 34 years old, and, therefore, had submitted to the operation at the age of 24. The spinal cord was removed by M. Laurent (assistant of M. Oulmont), and sent to me by the advice of my colleague, M. Verneuil.

Before placing the nerve in a weak solution of chromic acid, some layers of arachnoid fibres were noticed, upon the posterior face of this organ. There was also a little congestion, and, perhaps, thickening of the inner surface of the spinal dura mater, upon the posterior part of this envelope, at the level of the cervical region.

Transverse sections, made at different intervals of the cord, in its fresh state, showed to the naked eye, that the gray substance was less voluminous, on the left than on the right side, in the inferior part of the dorsal region, and in the dorso-lumbar enlargement. The white fasciæ, and particularly the posterior fascia of the left side, were in a similar condition.

When the spinal cord was sufficiently hardened by the chromic acid, incisions were made at different places; and examination by the microscope showed very perceptible diminution in the size of the left posterior fascia, and which—especially marked at the level of the dorso-lombar enlargement—was still apparent throughout the dorsal region, as far as the cervical enlargement. Even there, the same difference appears, in a slight degree, between the two posterior fasciæ, in the entire length of this enlargement; it is not apparent in the remainder of the cervical region, as far as the Rachidien bulb. Incisions made in the terminal cone of the dorso-lumbar enlargement still exhibit the same modifications, when not too far removed from the centre of the enlargement; but the resemblances in the two halves of the nerve increases in proportion to the approach to the lower extremity of the cone.

Throughout the altered portion of the spinal cord, and especially at the level of the dorso-lumbar enlargement, the difference between the white substance of the two sides is not restricted to that pertaining to the posterior fasciæ. Exact measurement shows that the left lateral fascia is not so thick as the one on the right. The anterior fasciæ present a nearly equal surface of extension, especially in the portion comprised between the anterior furrow and the internal boundary of the anterior cornu, and also in the dorso-lumbar region.‡

The gray substance, in the dorso-lumbar enlargement, presents a smaller surface of section on the left than the right. The difference extends through all portions of this substance, the anterior as well as the posterior cornu, and the intermediate tissue. The posterior cornu always exhibits the greater reduction of volume, as it is much smaller on the left than on the right side. The gray substance seems to extend equally on either side, as far as the dorso-lumbar enlargement.

Finally, in these modified portions, especially in the dorso-lumbar enlargement, the left half, regarded as a whole, and no longer considered in detail, is smaller than the right half. This difference is immediately apparent, upon an examination of a transverse section of the entire cord, in the region under consideration.

‡ It is necessary to state, that under some incisions, the left fascia was a little larger near the anterior commissure than the right anterior fascia, at the same level. (Fig. I. A.) The difference between these fasciæ was inverse to that existing in the posterior fasciæ; but it was less decided, and did not appear at all sections.

The granular appearance of the nervous fibres is greater, usually, in the left than in the right posterior fascia; it is so, at least, in the region of the gray commissure. The nervous-tissue is relatively more developed on the left than the right, in consequence of the diminished calibre of certain nerve-fibres, and, doubtless, of the disappearance of others. This tissue even appears to have experienced a slight hyperplasia.

There are no appreciable histological changes in the gray substance. The nerve-cellules of the different groups are recognized on the right and left. I have thought, under certain incisions, that these cellules were less numerous on the left than the right, especially in the external lateral group of the dorso-lumbar region. The vessels of both sides exhibit upon comparison the same characteristics in diameter and in structure.

The nerve-roots of the two sides, which are related by origin or termination to the dorso-lumbar enlargement, present the same appearance to the unaided eye. They are almost alike in size, and after maceration, in a solution of chromic acid, have the same color. Now, it is known that when the nerve-roots are atrophied, they take a lighter tint in this solution than when they are in a healthy state. Moreover, they have been examined by means of the microscope; and it has been impossible to ascertain from that any real alteration, such as the disappearance or the diminution in calibre, of the nerve-filaments, with or without granulations.

With the exception of what has just been said relative to the roots of the nerves, this observation coincides very nearly with those of M. Dickinson. In this case, as in those which he has examined, the gray substance and the posterior fascia were especially atrophied. In the following observation, the changes in the portion of the cord, corresponding to the amputated member, were far less apparent than in the instance just related.

Obs. II. Mr. S., aged 84 years, admitted to the hospital of Bicêtre, had an attack of apoplexy on the 6th of September, 1869. This was followed by hemiplegia of the right side. He died September 16th, in the care of M. Luys. There was found softening of the cerebral substance, on the left side, coinciding with the obstruction of the corresponding sylvian artery. This man has submitted to the amputation of the right thigh, near the inferior third, in 1853; probably in consequence of a fracture of the femur, occasioned by a fall from a vehicle. He was then 68 years old. Besides this, there was anchylosis of the elbow, on the same side. These particulars have been given me by M. Troisier, interne to Bicêtre, when he sent me the spinal cord of this man. It appears, also, that they found at the autopsy a small cavity (softening?), in the left half of the protuberance; and this half had diminished in size, especially below the cavity. The atrophy was not apparent in the Rachidien bulb. There were numerous layers of arachnoid fibres upon the posterior surface of the spinal cord.

After macerating the cord in a weak solution of chromic acid, for a month, sections made in different parts of the organ showed only a trifling difference in the two halves of the dorso-lumbar enlargement. Meanwhile, in this enlargement, and towards the inferior border of the dorsal region, the right posterior fascia is certainly a little smaller than the one on the left. The gray substance presents the same form and dimensions on both sides. The two halves of the cord resume their similiarity, in the greater part of the dorsal region, The difference between the two portions reappearing directly above the dorsal region extends as far as the cervical enlargement; but there the right anterior fascia is perceptibly smaller than the left. There is no more disparity between the anterior fasciæ, in and below the cervical enlargement. The nerve-tissue, there, is a little more developed in the posterior median granular fasciæ, particularly upon one side, than in its normal condition.

In the points where we have discovered a diminution of volume, either of the posterior or the anterior fascia of the right side, the substance of the nerve-tissue is likewise more abundant. But it is difficult to decide whether there is really an increase of the elements of this tissue, or whether it is more condensed by the disappearance or diminution of the nervous fibres. The intra-medullary vessels are decidedly larger than in the normal state, but they do not differ on the right and left.

We would state, in conclusion, that an innumerable quantity of amyloid bodies are found throughout the dorsal region of the cord, and that they are more numerous probably on the right than on the left, toward the lower part of this region. These amyloid bodies are seen most plainly in the posterior fasciæ, and in the posterior part of the lateral fasciæ. A considerable number exist also in the atrophied fascia of the portion immediately above the dorsal region.

It is evident that the modifications revealed in this instance are much less decided than in the previous case, or in the observations already published by M. Dickinson and myself.

To account for this difference, it is necessary to take into consideration the time which had elapsed between the date of the amputation and that of death, and especially the advanced age of the patient, when the amputation was performed. Now, in our last example, a man, 68 years of age, at the time of the operation, died, 16 years afterward. In the first case, it is true death occurred, only 10 years after the operation, yet the patient was but 24 years old when the thigh was disarticulated. The woman mentioned in the first observation, that I published last year, had suffered amputation of the right limb, at 12 years of age, and died 47 years after the operation. In the second case, there was doubt concerning a woman who was 39 years old * when her left limb was amputated, and who died 20 years later.

M. Dickinson, in his first observation, says, the patient was 21 years old when the amputation of the thigh was performed; death ensued 23 years after the operation. In the other two cases, the age is not given. The individuals each lived 23 years after the amputation of an arm. From the comparison of these data, it is evident at once, that the slight modifications described in my second observation, should be attributed especially to the advanced age of the subject at the time of the amputation.

* By an oversight, the age was not given in the note published last year on this subject. The woman had suffered amputation of the left limb, when 39 years old, and died of pneumonia at the age of 59.

The atrophie of the right anterior fascia, which was quite apparent in the upper part of the dorsal region and as far as the cervical enlargement, was certainly not occasioned by the amputation of the thigh, while, at the same time, the anterior fascia preserved its normal appearance, in the greater part of the dorsal region, and in the dorso-lumbar region. Should this be attributed to the diminution of the movements of the right arm, in consequence of the anchylosed elbow? In the absence of demonstrated facts, this question cannot be answered.

The first of the two new illustrations which I publish to-day, confirms, in many respects, those I have already made known, and those for which we are indebted to M. Dickinson. As I have already remarked, it is more nearly allied to the cases observed by M. Dickinson, than to those which I have examined. It is true, that in my observations, also, the posterior fascia was the portion of the white substance most atrophied. But I have not seen, as has M. Dickinson, decided alterations in the posterior nerve-roots, and, consequently, I am scarcely inclined to consider the entire atrophy of the posterior medullary fascia, simply as the direct and inevitable consequence of the posterior roots of the nerves, destined to the part amputated.

Notwithstanding the notable differences existing in the observations just published, they at least concur to demonstrate that the amputation of a limb occasions modifications in the gray and white substance of the spinal cord, and that these modifications are greatest in the portion of the cord which is related to the nerves of the amputated limb.

Since I first studied this subject, I have been desirous to know whether the simple section of the principle nerves in the limb of an animal would produce similar results. But it is only within a few months that I have been able to make this investigation.

M. Philipeaux, at my request, severed the sciatic and the crural nerves, in young rabbits, and in a full grown rabbit. I will give only three of the experiments, made on the young animals.

Exp. I. On the 26th of February, 1869, a section of the sciatic, and a branch of the crural nerve, of the right side, was made upon a rabbit, about a month old. The animal died, April 1st, 34 days after the operation. The reunion of the ends of the sciatic nerve was already well effected; and in the periphery, among the numerous fibres still altered, there were found many which were restored, and which possessed about half the diameter of the normal filaments.

The spinal cord having been hardened in a solution of chromic acid, thin layers of the organs removed at different distances were examined. Unfortunately, the maceration in the chromic acid rendered the nerve brittle, and it could not be satisfactorily examined. It may be stated, meanwhile, there were a few more granulations in the posterior fascia of the right side than of the left.

Exp. II. The same section was made in the left side of a rabbit, about a month old, June 3d, 1869. This animal died July 9th, 36 days after the operation. The sciatic nerve was found in the same condition as in the previous experiment.

The spinal nerve was examined, after hardening in a weak solution of chromic acid. The cord was preserved only in the lumbar and inferior dorsal region.

The nerve was first examined on the 12th of August, and incisions in the upper part of the lumbar enlargement showed that the posterior fascia of the left side was narrower than that on the right, and had even assumed a lighter color, while in the chromic acid, all the gray substance of the left side presented a lessened diameter. Other sections made in the cord still present this difference, although in a less degree. There can be no doubt regarding the disparity in size which exists in the posterior fasciæ, throughout the lumbar enlargement, and which is more evident at the level of the middle part of this enlargement than elsewhere. By taking comparative measurements, we ascertain that in some sections, the right posterior

We have employed the denominations applied to man, to indicate the regions and faces of the nerve, without reckoning the difference in the attitude of the animal.

fascia exceeds the left, in the ratio of 33 to 27, in others, 30 to 25, in others, still, 30 to 22. The difference is not so great below the enlargement, in the inferior portion of the dorsal region. The remainder of the cord was not preserved. The measurements just given were taken at a little distance from the commissure. The inequality between the two posterior fasciæ exists not only in their transverse measurement, but also in their antero-posterior dimensions. The fascia of the right side is perceptibly larger in front than that of the left. These conditions almost always exist in cases of this nature.

There is no marked dissimilarity in the two anterior fasciæ. A little difference is apparent near the anterior commissure; but this is of an opposite character to that existing in the posterior fascia. In all the sections, the left anterior fascia is larger than the right fascia, at the same point.

Exp. III. The same experiment (section of the sciatic nerve and one of the principal branches of the crural nerve) was made in the right side of a young rabbit. The animal died, June 17th, 1869, 50 days after the operation. The inferior portion of the cord (dorso-lumbar region) was put in alcohol for three days, and then in a weak solution of chromic acid. In this instance, the difference between the two halves of the cord is much more distinct than in the preceding cases. A section made near the upper part of the lumbar enlargement shows that the posterior fascia of the right side is smaller, by nearly one-half than that on the left; the ratio between the two fascia being that of 11 to 20. There is also considerable inequality in the two posterior cornua of the gray substance; measured in the greatest length, that of the left side corresponds to 28 divisions of the micrometer, while the one on the right corresponds to only 25 divisions. The anterior cornua of the antero-lateral fasciæ were of nearly the same dimensions, on the right and left. There is no apparent augmentation of the connective tissue, in the diminished posterior fascia. The decrease in the volume of this fascia is attributable mainly to the retraction of the nerve filaments, upon themselves. In fact, the diameter of many of these is smaller than the fibres in the corresponding fascia. The number of the nerve cellules is the same on both sides, and their histological characteristics are alike. In the inferior terminal cone of the spinal cord, the difference between the two halves grows less, and disappears entirely at a certain distance from the middle of the lumbar enlargement.

Exp. IV. The following experiment resembles the preceding ones, but was made upon a full-grown rabbit. The section of the large branch of the sciatic nerve, and of the crural nerve, of the right side, was made July 14th, 1869. The animal died, October 3d, 2 months and 19 days after the operation. An examination of the spinal cord, at the dorso-lumbar region, and more particularly of the lumbar enlargement shows, that the right posterior fascia is a little narrower than the left, and the furrow of separation curves a little toward the left fascia.

The two posterior fasciæ present less inequality than in the former experiments; where the difference is greatest, the right fascia exceeds the left, in the proportion of 30 to 25. The preceding experiments present the same phenomena that are observed in the individual, after the amputation of a limb. They show, as well, that the modifications in the spinal cord are more rapid and more extended, in proportion to the youth of the subject and the time intervening between the operation and the event of death. When the individual is very young—taking, for example, a rabbit one month old—a striking inequality can be observed in the two posterior fasciæ, even at the termination of 34 days. If the subject is grown, the modification of the posterior fascia, on the side of the several nerves, is less advanced at the end of two months and a-half than it was at the expiration of a month, in the young animals. Observations of the human subject and the experiments made upon animals show, that the arrest of development is not the only change taking place. Indeed, the fourth experiment and the cases of amputation, mentioned in this article, relate to adult individuals, the majority of whom had passed long ago the period of development. And in these cases, the atrophy of the nervous fibres could be ascertained. Upon comparison of the two sides of the cord, and after the assurance that one-half presents a smaller surface of section than the other, it is apparent that the posterior fascia of the atrophied side does not contain so many large nerve-filaments, as the posterior fascia of the opposite side. Moreover, the connective tissue is sometimes less developed there. Thus, as I said in regard to one instance, it is difficult to decide whether there is a real increase of this tissue, or whether this modification is not attributable, mainly, to the disappearance or the diminution of numerous nerve-filaments, the nerve being retracted, condensed, as it were, in consequence of this alteration.

The diminished number of the large fibres is a decisive indication, that in these conditions, a veritable work of atrophy had taken place.

But does this work of atrophy comprehend all?

We have seen that in the young subjects, rabbits, one and two months old, for example, the difference between the posterior fasciæ is already considerable, 34 days after the section of the sciatic and the crural nerves, of one side. †

Now, we can suppose, that in these cases, there had been not only atrophy of the nerve-filaments already existing, but even relative arrest in the development of the nervous elements of the fascia, corresponding to the several nerves. And even in the adults, if it is true, that during the whole period of life, some elements are gradually atrophied, while others develope, it is possible that this work of slow renovation may be more or less restricted, in different parts of the cord, in consequence of the section of the nerves, which originate in that region. It must be always understood, that these hypothesis are yet to be demonstrated, and that the only thing clearly established is the atrophy, which appears alike in the young and old.

It is impossible to obtain satisfactory conclusions, from examining the mechanism of this atrophy. The supposition

† In nearly all the experiments upon rabbits, it is certain that the entire crural nerve is not severed, and also that one of the branches of the sciatic nerve is intact. The modifications of the corresponding half of the spinal nerve would have been still more apparent if the section of the nerves had been complete.

immediately presenting itself is, that the atrophy of the posterior fasciæ is due to a work of ascending degeneration, occasioned by a functional inertia of the several nerves; the morbid process, starting from the place of section, invades, one after another, the upper extremity of the nerves, then the posterior medullary fasciæ, then the gray substance itself.

This is the supposition which M. Dickinson considers probable; and it is grounded upon the alteration which he has observed in the superior extremity of the nerves severed, in the cases of amputation, or in the nerve-roots connected with the modified region of the spinal cord.

I by no means doubt the accuracy of M. Dickinson's observation, but I have found nothing similar in the cases of amputation, in which I could examine the nerves of the stump, or the nerve-roots relating to them. The same result appeared in numerous cases of resection of the different nerves, the sciatic among others, observed in animals or in man. In these cases, when examining the central part of the nerves, at a little distance from the place of section, I have never found any alteration in the fibres, analagous to that displayed in the periphery. There were no granular fibres, and what I have said regarding a case of excision, of a segument of the sciatic nerve, in man, can best express the result of my investigations on this subject. In fine, if the modification of the superior extremity of the sciatic nerve really does exist, it is merely a diminution in the diameter of some nerve-fibres. ‡

I believe, then, that the alterations M. Dickinson describes are exceptional, and, consequently, they cannot furnish an explanation applicable to all cases.

Without admitting the degeneration noticed by M. Dickinson, the hypothesis of atrophy, by functional inertia, can still be sustained, the atrophy being limited to a diminution in the nerve filaments. But as the number of fibres thus affected varies considerably, it is doubtful whether one such modification suffices to account for the atrophy of the corresponding half of the cord, in the region connected with these nerves; an atrophy

‡ *Archives de Physiologie Normale et Pathologique*, 1869, p. 560.

which is, on the whole, quite extensive. Even the distribution of the atrophy prevents one from accepting this interpretation, as being fully satisfactory. Indeed, if I have alluded more frequently to the atrophy of the posterior fascia, it is not because this was the only atrophied part of the cord, but simply that the modification was greatest there, in the cases delineated in this paper. But there was also a diminution in thickness throughout the gray substance, in the anterior, as well as the posterior cornu, although it was more abundant in the former. Besides, the white substance of the lateral fascia was also thinner than in the normal state. Now, how can these different modifications be derived exclusively from a partially ascending atrophy of the severed nerves and of their spinal roots? Strictly speaking, the diminished volume of the posterior fascia and of the corresponding cornu may be explained thus. We can understand even the atrophy relating to the anterior cornu, if we admit there is an ascending modification, in the motive, as well as in the sensative, fibres. The anterior fascia, in this hypothesis, would remain exempt, or nearly so, because the fibres of the anterior root do not contract, in direct continuity with this fascia. But why do the laterial fasciæ present a similar diminution in thickness. It is true, this question can be answered by admitting that the motive and even the sensative fibres, after penetrating the gray substance, become a part of the lateral fasciæ, and that the atrophy of a considerable number of these fibres must occasion a diminution of volume in the corresponding lateral fascia.

However this may be, the explanation does not satisfy me completely. It does not account for such cases as I described last year, where the greatest atrophy existed in the anterior fascia. On the other hand, if the atrophy in the fibres of the nerve severed by amputation, in man, and by experimental section, in animals, is caused by the absence of the function of the nerve, I do not understand why only a small portion of these fibres are atrophied, while the great majority preserve, almost entirely, their normal characteristics, for a period of months, and even of years.

It is just as difficult to comprehend how an atrophy of this nature can be developed so rapidly in young animals. It has already been shown, that when the nerves of a lower limb have been severed, 34 days suffice to occasion a decided modification in the posterior fascia, and in the gray substance of the corresponding side. From these hypothesis, might it not be inferred, that the absolute functional repose of a nerve occasions, at its point of origin or termination, in the gray medullary substance, a physiological modification, followed, after a time, by a histological modification, which would determine the point where the atrophy of different parts of the spinal nerve would disappear?

Or would not the irritation, occuring in the severed nerves during the work of reparation, and which frequently exists long afterwards, limit the atrophy of the spinal cord, by its reaction upon that organ? The supposition that the process of atrophy begins in the gray substance gives no better explanation of the extensive ascending propagation, which takes place in the posterior fasciæ involved. I remember cases of amputation, in which this fascia, atrophied at the level of the dorso-lumbar enlargement, was also smaller at the superior dorsal region, than the fascia of the opposite side.

Before drawing any definite conclusion from these different hypothesis, it will be necessary to compare them with a number of facts additional to those already in our possession. The points which, I think, first merit especial attention are, the condition of the roots of the severed nerves, and of the nerves themselves, the histology of the gray substance, the number and form of the nerve-cellules, the condition of the nerve-tissue, etc. So long as contradictions and doubts remain, on these points, it will be impossible to form any legitimate theory of these modifications in the spinal cord.

Let us say, in conclusion, that these results, both of the clinical observations and the experiments mentioned in this paper, which show that perceptible modifications have immediately followed the section of the nerves, afford some light upon the important fact discovered by M. Brown-Séquard,* that epilepsy can be produced by severing the sciatic nerve. M. Brown-Séquard has made this application of the investigations referred to, while I presented a recapitulation of this work to the Society of Biology.

Just before sending this article to the press, I received from M. Trosier another spinal nerve, belonging to a man who died at Bicêtre. At the Hotel Dieu, on the 28th of May, 1868, this man submitted to the amputation of the left fore-arm, in the middle third. The operation performed by M. Langier was necessitated by a severe wound, from a fish-hook, penetrating the metacarpo phalangal articulation. In the month of January, 1869, the axillary glands became enlarged and painful. The patient being pronounced incurable was sent to the Hospital of Bicêtre, in the month of June. He was placed in the infirmary, under the charge of M. Marcxée; and, at this time, all the axilla was invaded by an epithelial production. He was constantly confined to his bed, and experienced great suffering in the axillary region. The cachexia gradually increased, and the patient died October 13th, 1869, six months and a-half after the operation, aged 48 years.

I can only make a superficial examination of this cord, which has been in a weak solution of chromic acid, only three or four days; but I can substantiate what M. Tozier has stated, in regard to the organ in its fresh state. The left anterior fascia is evidently smaller than the right, at the level of the cervical enlargement, while the dimensions of the posterior fasciæ seem nearly equal. The atrophy of the left anterior fascia is quite plain, in almost the entire length of the dorsal region. It is not easily recognized in the dorso-lumbar enlarge-

* I must repeat here what I have said elsewhere (*Societie de Biologie*), relative to a note which I published in the *Archives de Physiologie*, 1869, No. 2, p. 297: *Epilepsy observed in a guinea-pig, which had suffered section of one of the sciatic nerves.* I had observed this occurrence many years before publishing it, but without perceiving the relation existing between the section of the nerve and epilepsy. M. Brown-Séquard, to whom I related this case, and who had already observed similar ones, immediately showed me its signification, and requested me to insert it in our "*Recueil de Faitz.*"

ment, it exists in the entire extent of the cervical enlargement, as far as the immediate vicinity of the Rachidien bulb. In the upper part of the dorsal region, the modification of the left half of the cord is almost as great as in the cervical region. In the portion of the dorsal region alluded to, it is easily seen that the left anterior cornu of the gray substance is smaller than the right, in the proportion of 14 to 18. The difference between the anterior fasciæ is still more apparent, being in the ratio of 13 to 20. The two halves of the cord are by no means equal, when compared with each other; the right half exceeds the left in the proportion of 53 to 48, measured from the middle of the central canal, to each extremity of the transverse diameter of the cord. I will add, that neither in the cervical, nor superior dorsal region, has there appeared, from these early experiments, any thickening of the nerve-tissue, forming a part of the atrophied anterior fascia. We shall make further examinations on this point, when the cord is sufficiently hardened. The nerve-roots, at the level of the cervical enlargement, present no appreciable modification, to the unaided eye; the microscope gives the same negative result.

This new case accords with those I published last year, in which the anterior fascia was the most atrophied part of the white covering of the spinal cord. Also, it evinces, that in long standing cases of amputation, one can find, sometimes, the anterior fascia; but, doubtless, more frequently the posterior fascia of the same side, presenting a greater atrophy than the other parts. There is one grave difficulty, in addition to those just mentioned, and which can only be solved by the aid of more numerous and complete observations, with details of the pathological anatomy, as well as particulars relative to the physiological state of the amputated member, either before or after the operation.

